# Aflibercept for Wet Age-Related Macular Degeneration: A Prospective, Randomized Trial Comparing Treat-And-Extend and Fixed Bimonthly Dosing

**DOI:** 10.3390/jcm14228180

**Published:** 2025-11-18

**Authors:** Kevin Y. Wu, Shu Yu Qian, Alexandre Camiré, David T. Kim, Michel Giunta

**Affiliations:** 1 Department of Surgery, Division of Ophthalmology, University of Sherbrooke, Sherbrooke, QC J1G 2E8, Canada; 2 Faculty of Medicine, University of Sherbrooke, Sherbrooke, QC J1H 5H3, Canada; 3 Department of Family Medicine, University of Sherbrooke, Sherbrooke, QC J1H 5H3, Canada; 4 Department of Ophthalmology, University of British Columbia, Vancouver, BC V5Z 3N9, Canada; 5 Clinique GoGiunta, 20 12e Avenue S, Sherbrooke, QC J1G 2V4, Canada

**Keywords:** age-related macular degeneration, aflibercept, treat-and-extend, fixed dosing, non-inferiority

## Abstract

**Background/Objectives:** Currently, treatments for age-related macular degeneration (AMD) consist of regular intravitreal injections that exert significant pressure on healthcare systems via their high labor costs and economic burden. To address this, our study’s goal is to propose new treatment protocols by comparing the efficacy of bimonthly fixed dosing aflibercept injections versus the treat-and-extend (T&E) approach for wet AMD. Secondary objectives included categorical best-corrected visual acuity (BCVA) changes, anatomical outcomes, treatment intervals, and adverse events. **Methods**: This study is a 1-year randomized, open-label, prospective trial that included 44 eyes from 44 patients, 32 in the T&E arm and 12 in the bimonthly arm. Treatment-naïve AMD patients with neovascularization were randomized to a bimonthly fixed dosing group or a T&E group after receiving 3 consecutive monthly aflibercept injections. Following the induction doses, retreatment intervals for patients in the T&E arm were adjusted based on a predetermined algorithm. **Results**: Over 12 months, mean BCVA improvements were 5.0 letters in the bimonthly group and 4.1 in the T&E group (*p* = 0.096 for non-inferiority test). On average, T&E patients received 9.6 injections compared to 7.7 for those in the fixed dosing group (*p* < 0.001). Anatomical outcomes were comparable in both treatment arms. **Conclusions**: In our trial, the T&E approach provided significant visual improvements, but did not meet the threshold for non-inferiority when compared to fixed bimonthly dosing. It was also unable to minimize treatment burden over the course of the first year of injections. Further research is required to optimize the T&E algorithm with aflibercept.

## 1. Introduction

Age-related macular degeneration (AMD) is the leading cause of legal blindness in adults over 65 years old in developed nations, profoundly impacting vision-related quality of life [1]. Wet AMD (wAMD) accounts for approximately 90% of severe vision loss associated with AMD due to choroidal neovascularization (CNV) that ultimately leads to macular scarring [2].

Historically, treatment options for wAMD, such as laser photocoagulation and photodynamic therapy, were limited in their efficacy, often only slowing vision loss without significant improvement in visual acuity (VA) [3]. The advent of intravitreal monoclonal antibodies targeting vascular endothelial growth factor (anti-VEGF) has transformed the management of wAMD, offering the potential to maintain or even improve VA [4]. However, the standard monthly injection regimen required to control disease activity imposes a significant burden on both patients and healthcare providers. This pressure exerted by the considerable costs and labor needed for these treatment regimens would rapidly overwhelm the healthcare system [5]. To address this, alternative regimens have been developed, including fixed extended dosing intervals, pro re nata (PRN), and treat-and-extend (T&E) protocols. These approaches aim to reduce the frequency of injections while maintaining disease control and optimizing visual outcomes [6].

Ranibizumab, the first intravitreal anti-VEGF agent approved for wAMD, demonstrated in the MARINA and ANCHOR trials that monthly injections significantly improve VA [3,7]. To address this unsustainable regimen, numerous clinical trials were initiated to assess the efficacy and safety of alternative treatment schedules. The PIER study, which employed injections every 3 months following a loading phase of 1 injection monthly for the first 3 months, showed inferior visual outcomes [8]. The PrONTO study, on the other hand, introduced a PRN approach, using optical coherence tomography (OCT) findings, VA, and dilated fundus exams to guide treatment decisions. This method achieved statistically similar visual outcomes to those in the MARINA and ANCHOR studies while requiring less than half the number of injections within the same time frame [9]. These results inspired the ensuing development of individualized T&E regimens, where injection intervals are gradually extended once disease stability is achieved or shortened upon relapse [10].

Aflibercept, the second anti-VEGF agent approved for wAMD, has also shown efficacy in less frequent dosing regimens. Different than previous monoclonal antibodies, it is a recombinant fusion protein that traps all VEFG isoforms and prevents their binding to their respective receptors and the subsequent activation of the angiogenesis cascade [11]. The VIEW-1 and VIEW-2 studies demonstrated that its 8-week dosing regimen following three monthly loading doses provided visual outcomes equivalent to both monthly aflibercept and ranibizumab treatment schedules over a 2-year follow-up period [6]. The CLEAR-IT 2 trial also demonstrated that aflibercept’s efficacy can be maintained with a relatively low injection frequency during a PRN phase after an initial fixed-dosing period [12]. The study evaluated five intravitreal VEGF Trap-Eye dosing regimens, including fixed dosing every 4 or 12 weeks during the first 12 weeks, followed by PRN dosing for the subsequent 40 weeks. Significant reductions in central retinal/lesion thickness achieved during the fixed-dosing phase (−130 µm at week 52 compared to baseline) were maintained during the PRN phase. Similarly, significant gains in best-corrected visual acuity (BCVA) were preserved at week 52. Remarkably, a mean of only two injections was required during the PRN phase, with a mean time to first reinjection of 129 days, and nearly 20% of patients required no additional injections after initial loading phase [12]. These findings suggest that aflibercept’s biological activity is sufficiently durable to support extended dosing intervals, particularly after an initial loading phase [13].

While the T&E approach has demonstrated potential to reduce treatment burden without compromising outcomes, existing evidence is primarily based on studies involving ranibizumab, with only a small number of clinical studies specifically evaluating its use with aflibercept. One of them, the ALTAIR study, followed patients undergoing T&E aflibercept protocols where treatment intervals were adjusted by 2- or 4-week increments. At the 1 year-mark, average VA improved by more than 8 Early Treatment Diabetic Retinopathy Study (ETDRS) letters [14]. Jaggi et al. (2020) evaluated the efficacy of T&E aflibercept treatments and recorded significant VA improvements [15]. Similarly, Maruko and colleagues (2020) reported good anatomical and functional outcomes using the T&E approach, in accordance with the results described in the aforementioned studies [16].

At the time of writing, few prospective, randomized clinical trials have directly compared the T&E regimen with a fixed dosing regimen for aflibercept, illustrating a knowledge gap needing to be filled. Notably, the AZURE study aimed to compare the efficacy of both protocols in patients who have already undergone at least 1 year of fixed dosing aflibercept. In this investigation, researchers found the T&E schedule to be noninferior to its fixed dosing counterpart in terms of BCVA changes while needing a small number of injections [17]. Thus, building on this existing evidence, the TAEWA (Treat-and-Extend vs. fixed dosing Aflibercept in Wet AMD) study is a 1-year, randomized Canadian trial designed to directly compare the efficacy of fixed bimonthly aflibercept administration and the T&E regimen in maintaining optimal visual outcomes, the first of its kind on treatment naïve patients. By addressing this lack of data, the TAEWA study provides insights to optimize treatment strategies and refine clinical decision-making for patients with wet AMD.

## 2. Materials and Methods

### 2.1. Study Design

The TAEWA study is a 1-year randomized, open-label, prospective trial conducted in Canada to compare the efficacy of two aflibercept treatment regimens for wAMD: fixed dosing every 8 weeks (bimonthly) versus the T&E approach. The study aims to evaluate visual and anatomical outcomes while assessing treatment burden. The primary outcome was the change in BCVA from baseline to week 52 as well as the total number of injections received during the study period. Secondary outcomes included proportion of patients gaining or losing ≥5, ≥10, or ≥15 ETDRS letters, as well as changes in central retinal thickness (CRT) and OCT-angiography findings. Treatment frequency and duration of treatment-free intervals applied in the T&E arm were also documented. Furthermore, safety outcomes and the advent of adverse events (AEs) were closely monitored.

The study complies with Good Clinical Practice guidelines and relevant regulatory requirements. The study protocol (Bayer-17991) was reviewed and approved by the Advarra Institutional Review Board, registered with the Office for Human Research Protections and FDA under IRB#00000971. This trial was registered on ClinicalTrials.gov under NCT04113538 and conforms to CONSORT guidelines. The approved protocol (Pro00019224, continuing review approval was granted on 23 December 2018) and informed consent form (Advarra IRB Approved Version, 22 May 2019) align with ethical principles outlined in the Declaration of Helsinki. Written informed consent was obtained from all participants before enrollment.

A total of 44 patients were recruited at a single site, GOGiunta’s clinic (Sherbrooke, QC, Canada), which has prior experience in wAMD studies and an established track record of recruiting treatment-naïve patients with wAMD. Enrollment was completed within 6 months, with the investigator and study team identifying eligible patients during routine clinical practice. The recruitment strategy ensured that participants met all inclusion criteria and provided written informed consent.

The inclusion criteria were:

- Treatment-naïve patients aged 50 years or older;
- Diagnosis of CNV secondary to wAMD, confirmed by fluorescein angiography (FA) and OCT;
- BCVA between 78 and 19 ETDRS letters (approximate Snellen equivalent of 20/32 to 20/400) in the study eye.

The exclusion criteria were:

- Advanced subretinal fibrosis or significant geographic atrophy involving the foveal center.
- Presence of severe ocular comorbidities in the study eye, such as uncontrolled glaucoma, diabetic retinopathy likely to be visually significant within 2 years, cataracts requiring surgery within 2 years, vitreous or preretinal hemorrhage obscuring the central macula, or rhegmatogenous retinal detachment.
- Clinical suspicion of polypoidal choroidal vasculopathy.
- Active or suspected ocular or periocular infections in either eye, or active intraocular inflammation in either eye.
- Known sensitivity to aflibercept or any component of its formulation.
- Recent history of new-onset cardiac disease or thromboembolic events within 12 months prior to baseline, or systemic treatment with anti-VEGF therapies within 60 days prior to enrollment.
- Any prior treatment in the study eye, including bevacizumab, ranibizumab, verteporfin, dexamethasone, external radiation therapy, transpupillary thermotherapy, or any other intravitreal injection.
- Concurrent participation in another clinical trial or participation in a clinical trial within 30 days prior to enrollment.
- Any condition that, in the opinion of the principal investigator, could significantly impact treatment assessments during the study.

### 2.2. Randomization and Treatment Arms

Patients were randomized in a 2:1 ratio into the T&E arm or the fixed bimonthly dosing arm using an online randomization tool. Randomization occurred at week 8 after the loading phase. In the fixed bimonthly dosing arm, patients received three monthly loading doses of aflibercept (2 mg) followed by fixed injections every 8 weeks for the remainder of the study. In the T&E arm patients, received the same loading phase as the fixed-dosing group. Following stabilization, injection intervals were adjusted based on disease activity. Criteria for adjusting intervals included the presence or absence of fluid, changes in BCVA, central retinal thickness (CRT), and anatomical findings such as new neovascularization or hemorrhage. Intervals could be extended or reduced by 2- or 4-week increments, for a minimum of 4 weeks and a maximum of 12. The algorithm for determining retreatment intervals is provided in Table 1.

Participants underwent a screening visit, where eligibility was assessed through BCVA measurement, OCT, FA, and other ophthalmic evaluations. Baseline assessments included detailed imaging. Follow-up visits were conducted at each injection appointment and included BCVA assessment using ETDRS charts, CRT measurement via OCT, and additional imaging (fundus photography, OCT-angiography, and FA as needed). The final follow-up occurred at Week 52, with comprehensive evaluations including BCVA, CRT, FA, and patient-reported outcomes. BCVA was measured at each visit using standardized ETDRS charts at a 4-m distance. Contrast sensitivity was evaluated to measure the ability to discern differences between light and dark. OCT and OCT-angiography were performed at each visit to assess CRT and vascular changes. Fundus photography and FA were performed at screening, Month 12, and as clinically indicated to evaluate retinal vasculature. Slit-lamp biomicroscopy, intraocular pressure (IOP) measurement, and indirect ophthalmoscopy were also conducted at each visit to monitor anterior and posterior segment health.

### 2.3. Statistical Analysis

All variables were analyzed descriptively. Changes in BCVA, CRT, and other efficacy measures were analyzed using mixed-model repeated measures. Categorical outcomes, such as proportions of patients achieving significant BCVA changes, were analyzed using chi-square or Fisher’s exact test. Continuous data were evaluated with Student’s *t*-test or Mann–Whitney U test. All tests were two-tailed, with a significance level of 5%. Missing data were handled using the last observation carried forward method. Specific statistical resources to validate the reliability of mixed-model repeated measures were not utilized. To compare the therapeutic efficacy of both regimens, we performed a 2 sample non-inferiority test with a non-inferiority threshold of 5 ETDRS letters. Any patients who withdrew consent or were later found to be ineligible were excluded from our analyses. Likewise, patients withdrawn from the study before randomization were also excluded. All other patients, encompassing those that dropped out after randomization, were included in our full analysis set. Sensitivity analyses were performed by evaluating primary outcomes when incorporating and excluding individuals who did not complete the full study period to ensure robustness. Data were presented as frequencies or means ± standard deviation (SD). Statistically analyses were performed using R v.4.3.1 (R Core Team, Vienna, Austria).

## 3. Results

### 3.1. Patient Enrollment

**Figure 1:**
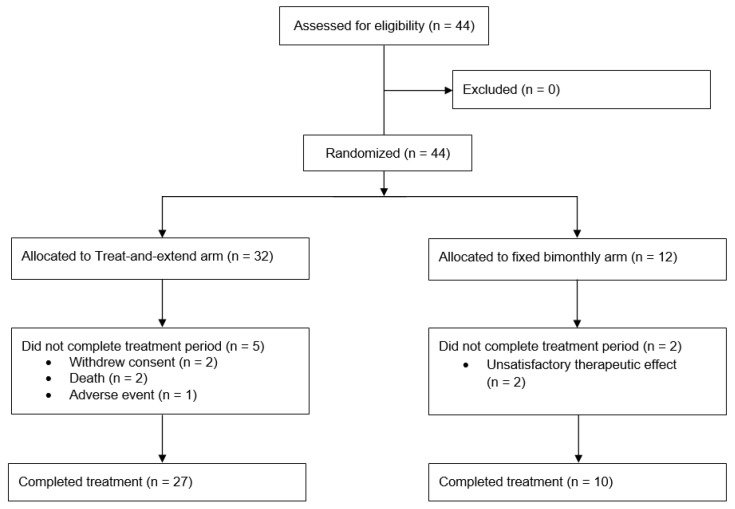
Consort diagram illustrating the flow of participants at each stage of the trial.

Between 10 September 2019, and 5 January 2022, a total of 44 patients were recruited for the TAEWA study, with 32 assigned to the T&E group and 12 to the bimonthly group. Of these, 7 patients (5 in the T&E group and 2 in the bimonthly group) did not complete the full treatment course and were excluded from statistical analysis. 2 patients were excluded because they withdrew consent and 3 discontinued the study early due to treatment-emergent adverse events (TEAE). The study was terminated early for the remaining 2 patients due to unsatisfactory therapeutic responses. The safety analysis set (SAF) excluded the 2 withdrawals while the full analysis set (FAS) excluded an additional 2 patients who succumbed to illnesses early in the study period before the randomization step occurred (2 of the 3 patients having TEAEs). Thus, the SAF contained 42 patients, and the FAS had 40. After accounting for all exclusions, 37 patients (27 in the T&E group and 10 in the bimonthly group) ultimately completed the treatment, as detailed in Figure 1. The exclusion of those who did not complete the entire was statistically addressed by performing sensitivity analyses that included patients who suffered TEAEs or experienced unsatisfactory therapeutic effects. Our primary outcomes were computed both with and without these excluded individuals to evaluate robustness. These analyses confirmed that primary outcomes were comparable and that conclusions remained identical despite these exclusions.

### 3.2. Baseline Characteristics

Baseline demographic and disease characteristics were comparable between the two treatment groups. All participants in our trial were of Caucasian ethnicity. The mean age of participants was 80.2 ± 4.4 years in the fixed dosing group and 79.3 ± 6.5 years for the T&E group. 8 patients (66.7%) in the bimonthly dosing group were female compared to 15 (53.6%) in its T&E counterpart. Baseline demographics were similar in both groups, but statistically significant differences were present in terms of disease characteristics. Baseline mean CRT was 311.9 ± 82.4 µm in the T&E arm and 377.7 ± 104.2 µm in the bimonthly arm. Initial BCVA was 65.9 ± 12.6 letters in the T&E group and 54.9 ± 17.8 letters in the bimonthly group. These characteristics are detailed in Table 2.

### 3.3. Primary Outcomes

**Figure 2:**
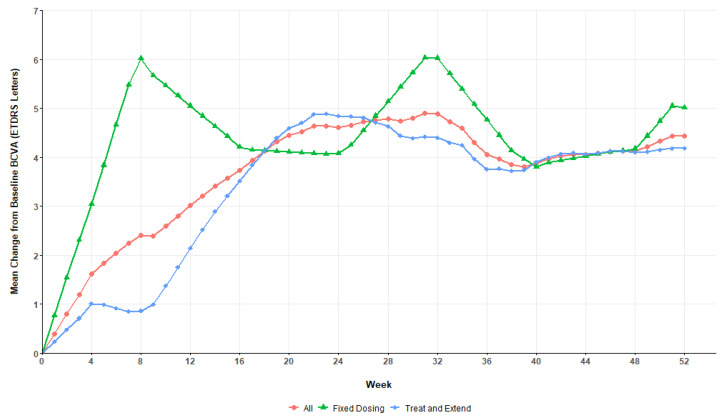
Mean change in BCVA by treatment arm from start to 52 weeks. Full set analysis, last observation carried forward.

The primary analysis evaluated the change in BCVA from baseline to the measurement at week 52. By the end of the follow-up period, the mean change in BCVA was 5.0 ± 8.4 (95% confidence interval [CI] 0.2–9.8) letters in the fixed dosage arm and 4.1 ± 12.3 (95% CI −0.5–8.6) letters in the T&E arm. The T&E group also had the widest range of BCVA variations with one patient gaining 36 letters while another patient lost 31. Using the prespecified threshold of 5 letters, statistical tests demonstrated that in our study, the T&E protocol cannot be deemed non-inferior to its regular bimonthly counterpart (*p* = 0.096). Figure 2 details the evolution of the mean BCVA in both treatment arms across the study period. On average, patients in the fixed dosing group achieved their best acuities after 8 weeks while the BCVA of their T&E counterparts only peaked around the half-year mark. After reaching a maximum, patients’ BCVA generally stabilized around that value until the end of the 52 weeks.

**Figure 3:**
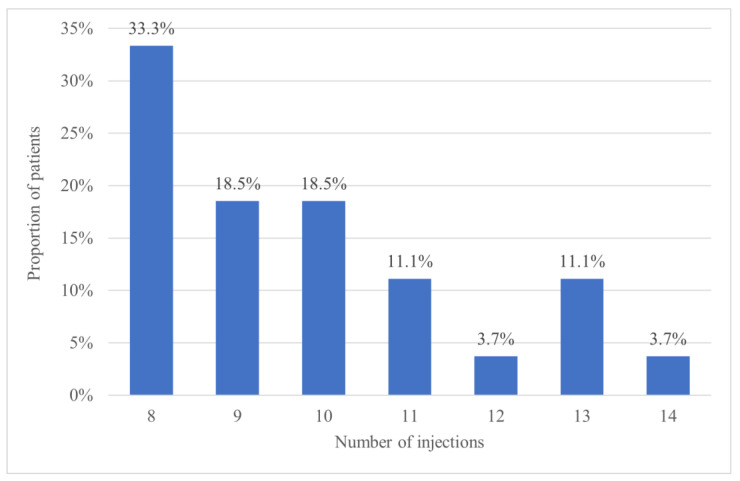
Total number of injections over 52 weeks for patients in the treat-and-extend group.

Over the entire study period, the mean number of injections was 9.6 ± 2.0 (95% CI 8.9–10.3) in the T&E arm and 7.7 ± 1.3 (95% CI 7.0–8.4) in its fixed dosing counterpart (*p* < 0.001), thus indicate that patients in the T&E group received significantly more treatments. The maximum number of treatments given in the bimonthly group was 9, while those on the T&E protocol received up to 14 injections. More specifically, 13 patients (48.1%) in the T&E group required 10 or more injections, as shown in Figure 3.

As a complement, additional analyses were conducted excluding all patients who discontinued the study due to adverse events or unsatisfactory therapeutic response. The conclusions of this analysis remained consistent with those of the primary analysis, indicating that the exclusion of these patients did not alter the overall findings.

### 3.4. Secondary Outcomes

**Figure 4:**
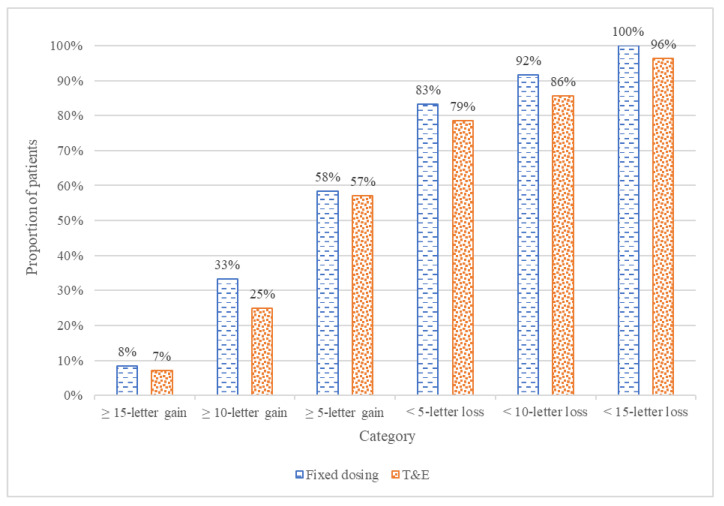
Categorical BCVA change for patients in both treatment arms.

The secondary analysis evaluated clinical variables derived from BCVA, which included the proportion of patients with a gain or loss of 5, 10, or 15 ETDRS letters. At week 52, the proportion of patients with a gain of 5 or more letters was nearly identical between the T&E and fixed bimonthly groups, with 16 (57%) and 7 (58%), respectively (*p* > 0.9). The bimonthly arm also had slightly higher rates of patients gaining ≥10 letters and ≥15 letters, although these differences were not statistically significant. By the end of the study, all but one patient maintained vision (<15-letter loss), with the one exception being in the T&E arm. Overall, the bimonthly arm had slightly higher proportions of patients across all subcategories, although none of these differences are statistically significant. These results are presented in Figure 4.

The CRT from baseline to the closest visit to week 52 experienced clinically significant decreases across both treatment arms. In the fixed bimonthly group, the mean change in CRT was −131.8 µm ± 104.1 µm. In the T&E group, that value was −103.7 µm ± 83.8 µm. This difference in the mean change was not statistically significant (*p* = 0.4).

Changes in OCT angiography (OCT-A) parameters during the study period were analyzed across both treatment groups. Three key variables were evaluated: changes in OCT-A dimension, central perfusion, and total perfusion. The mean reduction in OCT-A dimension was similar between groups, with both treatment groups showing identical mean reductions of 0.3 ± 0.9 mm. For central perfusion, the fixed bimonthly group showed a mean reduction of 8.4 ± 15.4%, while the T&E group demonstrated a slightly greater reduction of 9.8 ± 14.8% (*p* = 0.8). In the fixed bimonthly group, there was a mean increase of 5.7 ± 12.7% for total perfusion, compared to −0.8 ± 13.2% in the T&E group. Despite this notable numerical difference, it was not statistically significant (*p* = 0.2). In summary, the results indicate that the fixed bimonthly and T&E regimens produced comparable outcomes for all OCT angiography parameters analyzed, with no significant differences detected between the two groups.

We observed a statistically significant difference between the fixed bimonthly and T&E regimens in terms of treatment intervals over the entire study period. Notably, in the fixed bimonthly group, the mean interval was 6.8 ± 0.3 weeks, in accordance with the protocol-driven schedule. In contrast, the T&E group had a shorter mean interval of 6.1 ± 1.2 weeks (*p* = 0.008). The last mean treatment interval up to week 52 in the T&E group was 8.6 ± 2.7 weeks. For this final injection, 8 patients (28.6%) were able to space out their treatment interval to 12 weeks, which is the longest interval achieved by any patient during the entire follow-up period. On the contrary, 3 patients remained at 4 weeks at the end of the study, of which 2 did not have their interval extended a single time. Proportions of T&E patients achieving various final treatment interval lengths are shown in Table 3.

The number and proportion of injections administered outside the treatment window (defined as a range of 5 days before or after the protocol-specified treatment date) were analyzed for the overall population and across treatment groups. In the fixed bimonthly group, the mean number of injections out of window was 1.6 ± 1.6 injections, while the T&E group showed a higher mean of 2.8 ± 2.5 injection. This difference was not statistically significant (*p* = 0.073).

In this study, 3 patients, all in the T&E group, suffered TEAEs. 2 of them developed cancers (lung and pancreatic) and the remaining one suffered recurrent falls requiring hospitalization. None of these AEs were deemed related to aflibercept. Among the 360 injections given, we do report the occurrence of any ocular or systemic AEs such as increased intraocular pressure, uveitis, endophthalmitis, retinal tear, vitreous hemorrhage, cataract progression, or arterial thromboembolic events. However, none were observed during the course of this study.

## 4. Discussion

The enhancement of treatment protocols for wAMD patients has been of increasing interest to retinal specialists with the development of new treatment modalities. For aflibercept, several previous studies have shown its efficacy in T&E regimens, but few have offered direct comparisons with fixed dosing. To the best of our knowledge, our study is the first prospective, randomized clinical trial directly comparing both protocols in treatment-naïve wAMD patients. By addressing this gap in the knowledge, the TAEWA study provides insights into optimizing treatment strategies.

### 4.1. Comparison with Twin Studies

In our study, the T&E regimen failed to demonstrate non-inferiority to the fixed bimonthly regimen for visual acuity outcomes at week 52. Mean BCVA improvement was 4.7 letters in the fixed group and 4.1 letters in the T&E group, suggesting that the fixed regimen may provide slightly more consistent therapeutic benefit. Differing baseline clinical characteristics in our groups may potentially impact this outcome, as fixed dosing patients started off with worse mean VA. Given that patients with lower baseline VA often have more potential for improvement, this initial disparity may have contributed to the slightly greater BCVA gain observed in the fixed regimen group. Huang and colleagues’ analysis (2024) revealed that when treated with aflibercept, poor baseline VA strongly correlated with better visual improvement [18]. The results in our trial nonetheless contrast those from the AZURE study, where researchers compared both protocols in patients who have already received over 1 year of fixed dosing aflibercept injections prior to enrollment. Despite differences in methodology, their investigation found the T&E regimen to be non-inferior to its bimonthly counterpart with both treatments equally capable of maintaining vision over a 76-week period [17]. A retrospective study by Abdin et al. (2021) also found that over 1 year, the T&E and bimonthly arms provided statistically similar visual acuity improvements (7 and 10 letters respectively) [19]. In other reports examining T&E aflibercept regimens, the mean VA change varied from +1.3 to +11.8 letters after 12 months of treatments [14,15,20,21]. Our results thus fall toward the lower end of that range.

During the course of our study, the mean number of injections was significantly higher in the T&E group compared to the fixed bimonthly group (9.6 and 7.7 injections respectively). These findings highlight a greater treatment burden for our T&E patients and challenge the assumption that the T&E regimen necessarily reduces injection frequency. In our case, the initial loading phase of 3 monthly injections possibly contributes to this difference: the fixed dosing group automatically jump to 8-week intervals while T&E patients gradually worked their way up from 4 weeks. Specifically in our cohort, around half of all patients in the T&E arm needed 4-to-6-week injection intervals for the first 6 months before slowly being able to further space out appointments during the second half of the study period. For this outcome, Abdin and colleagues’ study concurs with ours, where their T&E patients also received significantly more injections on average than their fixed dosing counterparts (8.5 vs. 7.0 injections) [19]. The AZURE trial, on the other hand, reports differing results where the mean number of treatments in the T&E arm was 6.0 compared to 6.8 in the bimonthly arm. It is relevant to note, however, that AZURE started with all patients already being on 8-week intervals [17].

The secondary outcomes were mostly similar between treatment arms. Both T&E and fixed bimonthly regimens maintained visual stability, with similar proportions of patients achieving clinically meaningful gains or avoiding significant losses in BCVA. CRT reductions were also comparable between groups, indicating that both regimens effectively control disease activity at the anatomical level. Additionally, OCT angiography parameters showed no significant differences between groups, suggesting both approaches are similarly effective in stabilizing neovascular activity. These anatomical outcomes are therefore consistent with those presented in previous studies [14,17,19]. In our own study, the variability observed in OCT and OCT-A measurements may be partly attributable to the relatively limited sample size as our study was not powered based on these secondary outcomes. Additionally, inherent fluctuations in OCT and OCT-A parameters are well recognized among patients with neovascular AMD receiving anti-VEGF therapy, due to variable treatment response dynamics and disease activity over time [22].

Despite these similarities, the T&E approach demonstrated a significantly shorter injection intervals (aligns with the higher injection frequency observed), underscoring its increased treatment burden compared to the fixed bimonthly regimen. Experiments have calculated that intravitreal aflibercept has a median half-life of 9 days and that its VEGF suppression can last up to 16 weeks, hence demonstrating that it can theoretically tolerate the prolonged treatment intervals in T&E protocols [23,24]. In fact, previous trials have found that 37% to 50% of patients can achieve injection intervals of 12 weeks or greater after 1 year [14,17,20]. Our study did not obtain the same level of success potentially due in part to the differences in the protocol for treatment adjustments. Additionally, since the duration of aflibercept’s effects varies among individuals, intrinsic differences in patient groups across these studies may account for some of the disparity [24].

Aflibercept-related AEs are uncommon, with cataract development, dry eyes, increased intraocular pressure, and conjunctival hemorrhage being the most frequently reported ones [14,17,20]. Severe TEAEs such as endophthalmitis and thromboembolic events occurred in less than 2% of patients [14,17,21]. In our study, only 3 treatment-emergent complications were documented, and none of them were considered related to aflibercept injections. These findings are consistent with the previously known safety profile and raises no new safety concerns.

### 4.2. Future Directions

The TAEWA study provides insights into the comparative efficacy and practicality of the T&E and fixed bimonthly regimens for aflibercept. While the T&E approach has previously demonstrated success in reducing treatment burden with other anti-VEGF agents, its application to aflibercept may not consistently achieve this benefit, as evidenced by the higher injection frequency and lower visual acuity observed in this study. Despite not reaching the margin for non-inferiority, patients in the T&E group still achieved noticeable BCVA improvements, suggesting the viability of this approach. However, there is considerable variability across T&E regimens, and no formal consensus exists at this current moment. While fundamental treatment principals remain the same, the exact criteria for interval adjustments vary slightly between different investigations. Moreover, our study set a maximum interval length of 12 weeks, ARIES and ALTAIR capped it at 16 weeks, while AZURE had no upper limit [14,17,20]. The specific details of an ideal T&E protocol could merit further fine-tuning.

### 4.3. Limitations

Our study has several limitations. First, the single-center design, combined with the exclusively Caucasian patient cohort, may limit the generalizability of these findings to more diverse clinical settings and populations since disease progression and treatment responsiveness may vary across ethnicities. Notably, one investigation by Osathanugrah et al. (2021) revealed that Black patients had poorer outcomes relative to Caucasian and Hispanic individuals when treated with bevacizumab for diabetic macular edema [25]. These findings thus call into question the possibility of similar racial correlations when using aflibercept for wAMD. Another limitation of our study is the relatively small sample size (*n* = 40) as well as imbalances in baseline characteristics, which may have limited the statistical power to conclusively demonstrate non-inferiority between the regimens. Longer-term follow-up beyond 52 weeks is necessary to assess the durability of these outcomes and the long-term safety profiles of both regimens. Long-term data on the efficacy of T&E aflibercept remains sparse and inconsistent. So far, certain studies report maintenance of the letters gained while others documented that BCVA peaks after 12 months and slowly returns to baseline in subsequent years [14,16,20,21]. Therefore, larger, multicenter trials are needed to validate these findings in more diverse patient populations. Additionally, real-world data on resource utilization and patient-reported outcomes could offer insights to better assist clinical decision-making.

Due to the numerous aforementioned limitations such as small sample size, single-centered design, and differences in baseline characteristics between groups, it is challenging to pinpoint the exact reason behind the fact that our T&E approach failed to achieve non-inferiority. It is also a possibility that our specific T&E protocol may have been suboptimal for our patients and masked the benefits of this strategy found in previous reports. Our findings do not necessarily invalidate the advantages of the T&E regimen, but rather illustrate the presence of opportunities for enhancements. Accordingly, we thus encourage further research to refine the T&E approach with aflibercept, especially focusing on revising the algorithm for the determination of retreatment intervals.

## 5. Conclusions

The TAEWA study revealed that the T&E regimen did not demonstrate non-inferiority to the fixed bimonthly regimen in visual acuity outcomes, with the fixed regimen showing a slightly greater mean improvement in BCVA at week 52. Moreover, the T&E approach required significantly more injections, highlighting its higher treatment burden during the first year. These findings illustrate the potential limitations of the T&E regimen when applied to aflibercept and support the potential advantages of a fixed dosing schedule for more consistent and predictable outcomes. However, our results do not necessarily negate the benefits of a T&E approach as brought to light in previous studies. Rather, our conclusions may be influenced by the aforementioned limitations, such as small patient population, short study duration, and uneven distribution of baseline clinical features. Nonetheless, we believe that treatment effects are significant due to our standardized methodology and systematic treatment algorithms. As such, our data may also be utilized as a pilot study for future and more adequately powered studies. Moreover, future research could benefit from improved patient recruitment and adherence. Subsequent investigations should also aim to optimize T&E protocols and more conclusively demonstrate their long-term viability.

## Figures and Tables

**Table 1:** Algorithm for Determination of Retreatment Interval.

Condition	Criteria	Action
Optimal Condition	- No fluid - No loss of ≥5 ETDRS letters (from previous treatment visit) - No increase of ≥100 µm in CRT (compared to the previous lowest value) - No new neovascularization - No new macular hemorrhage	Extend treatment interval by 2 weeks
Improving Condition	- Residual but decreased fluid (compared to previous visit) - No loss of ≥5 ETDRS letters (from previous treatment visit) - No increase of ≥100 µm in CRT (compared to the previous lowest value) - No new neovascularization - No new macular hemorrhage	Maintain current treatment interval
Deteriorating Condition	- New fluid - Persistent unchanged or increased fluid - Loss of ≥5 ETDRS letters (from previous treatment visit) - Increase of ≥100 µm in CRT (compared with the previous lowest value) - New neovascularization - New macular hemorrhage	Reduce treatment interval by 2 weeks (if current interval is 6–8 weeks) or by 4 weeks (if current interval is 10–12 weeks)

**Table 2:** Patient demographics and baseline clinical characteristics.

Characteristic		Bimonthly	T&E	*p*-Value
Mean age (years)		80.2 ± 4.4	79.3 ± 6.5	0.6
Gender				0.5
	Female	8 (66.7%)	15 (53.6%)	
	Male	4 (33.3%)	13 (46.4%)	
Study eye				0.7
	Right	5 (41.6%)	14 (50%)	
	Left	7 (58.3%)	14 (50%)	
Smoker status				0.8
	Smoker	0 (0%)	1 (3.6%)	
	Ex-smoker	5 (41.6%)	10 (35.7%)	
	Non-smoker	7 (58.3%)	17 (61%)	
BCVA (letters)		54.9 ± 17.8	65.9 ± 12.6	0.02
CRT (µm)		377.7 ± 104.2	311.9 ± 82.4	0.03

Data are represented as number (%) or mean ± SD.

**Table 3:** Last treatment interval up to week 52 in the T&E group.

Treatment Interval	Number (%)
4 weeks	3 (10.7%)
6 weeks	5 (17.9%)
8 weeks	8 (28.6%)
10 weeks	4 (14.3%)
12 weeks	8 (28.6%)

## Data Availability

Data is contained within the article.

## Abbreviations

The following abbreviations are used in this manuscript:

AE	Adverse event
AMD	Age-related macular degeneration
BCVA	Best-corrected visual acuity
CNV	Choroidal neovascularization
CRT	Central retinal thickness
ETDRS	Early Treatment Diabetic Retinopathy Study
FA	Fluorescein angiography
FAS	Full analysis set
OCT	Optical coherence tomography
OCT-A	Optical coherence tomography-angiography
PRN	Pro re nata
SAF	Safety analysis set
SD	Standard deviation
TEAE	Treatment-emergent adverse event
T&E	Treat-and-extend
VA	Visual acuity
VEGF	Vascular endothelial growth factor
wAMD	Wet age-related macular degeneration
